# Doxycycline attenuates l-DOPA-induced dyskinesia through an anti-inflammatory effect in a hemiparkinsonian mouse model

**DOI:** 10.3389/fphar.2022.1045465

**Published:** 2022-11-23

**Authors:** Maurício dos Santos Pereira, Glauce Crivelaro do Nascimento, Mariza Bortolanza, Patrick Pierre Michel, Rita Raisman-Vozari, Elaine Del Bel

**Affiliations:** ^1^ Department of Basic and Oral Biology, FORP, Campus USP, University of São Paulo, Ribeirão Preto, Brazil; ^2^ Sorbonne Université, Paris Brain Institute-ICM, Inserm, CNRS, APHP, Hôpital de La Pitié Salpêtrière, Paris, France

**Keywords:** neuroinflammation, cytokines, tetracyclines, Parkinson’s disease, IL-1β, TNF-α, PGE_2_, COX-2

## Abstract

The pharmacological manipulation of neuroinflammation appears to be a promising strategy to alleviate l-DOPA-induced dyskinesia (LID) in Parkinson’s disease (PD). Doxycycline (Doxy), a semisynthetic brain-penetrant tetracycline antibiotic having interesting anti-inflammatory properties, we addressed the possibility that this compound could resolve LID in l-DOPA-treated C57BL/6 mice presenting either moderate or intermediate lesions of the mesostriatal dopaminergic pathway generated by intrastriatal injections of 6-OHDA. Doxy, when given subcutaneously before l-DOPA at doses of 20 mg kg^−1^ and 40 mg kg^−1^, led to significant LID reduction in mice with moderate and intermediate dopaminergic lesions, respectively. Importantly, Doxy did not reduce locomotor activity improved by l-DOPA. To address the molecular mechanism of Doxy, we sacrificed mice with mild lesions 1) to perform the immunodetection of tyrosine hydroxylase (TH) and Fos-B and 2) to evaluate a panel of inflammation markers in the striatum, such as cyclooxygenase-2 and its downstream product Prostaglandin E2 along with the cytokines TNF-α, IL-1β and IL-6. TH-immunodetection revealed that vehicle and Doxy-treated mice had similar striatal lesions, excluding that LID improvement by Doxy could result from neurorestorative effects. Importantly, LID inhibition by Doxy was associated with decreased Fos-B and COX-2 expression and reduced levels of PGE_2_, TNF-α, and IL-1β in the dorsolateral striatum of dyskinetic mice. We conclude 1) that Doxy has the potential to prevent LID regardless of the intensity of dopaminergic lesioning and 2) that the anti-inflammatory effects of Doxy probably account for LID attenuation. Overall, the present results further indicate that Doxy might represent an attractive and alternative treatment for LID in PD.

## 1 Introduction

l-DOPA-induced dyskinesia (LID) is a significant motor complication of dopamine (DA)-replacement therapy in Parkinson’s disease (PD) patients. Clinically, LID manifests as aimless irrepressible movements that unsettle the wellbeing of individuals (Cenci et al., 2020). Large intermittent oscillations in brain dopamine levels primarily account for LID pathophysiology by producing an anomalous stimulation of postsynaptic DA receptors, predominantly those expressed on striatal neurons (Kordower et al., 2013; Cenci, 2014; Olanow et al., 2020). Elevations in extracellular glutamate levels in the striatum and the *substantia nigra pars reticulata* that are also typical of this neurological condition, lead to neurochemical disturbances that contribute to the manifestation of abnormal involuntary movements (AIMs) (Sebastianutto and Cenci, 2018; Dos Santos Pereira et al., 2020).

The neurotransmitter imbalance in the basal ganglia of dyskinetic animals also leads to neuroinflammation, another critical hallmark of LID (Del-Bel et al., 2016; Carta et al., 2017; Pisanu et al., 2018; Morissette et al., 2022). Experimental data collected over recent years revealed, for instance, that pro-inflammatory enzymes, such as cyclooxygenase-2 (COX-2) and the inducible nitric oxide synthase (iNOS), are activated in the basal ganglia structures of rodents with l-DOPA-induced AIMs (Bortolanza et al., 2015a; Bortolanza et al., 2015b). Besides, astrogliosis and microgliosis have been described in the dorsolateral striatum of dyskinetic rodents (Barnum et al., 2008; Bortolanza et al., 2015a; Mulas et al., 2016; Dos Santos Pereira et al., 2020), and pro-inflammatory soluble factors such as TNF-α and IL-1β are elevated in this area.

Tetracyclines are well-known broad-spectrum antibiotics primarily described in the late 1940s (Chopra and Roberts, 2001). The importance of tetracyclines has been recently revisited, as some of these compounds have the potential to be repurposed for different pathological conditions. This is particularly true for doxycycline (Doxy), a compound that has the potential to treat several neurological and non-neurological conditions. The pharmacological control of chronic inflammation by Doxy seems to be fundamental for its benefits in cancer (Mortison et al., 2018), diabetes (Wang et al., 2017), autoimmune diseases (Park et al., 2020) and central nervous system (CNS) neurodegenerative pathologies (Orsucci et al., 2009; Bortolanza et al., 2018).

The neuroprotective effects of Doxy in animal models of PD indicate that this drug is a potential candidate for the treatment of this disorder. These effects are also primarily imputed to its anti-inflammatory properties (Griffin et al., 2011; Bortolanza et al., 2018). For instance, Doxy protects DA neurons from degeneration induced by 6-hydroxydopamine (6-OHDA) and 1-methyl-4-phenyl-1,2,3,6-tetrahydropyridine (MPTP) by concomitantly repressing gliosis (Cho et al., 2009; Lazzarini et al., 2013). Clinically, evidence of a lower incidence of PD in patients treated with Doxy for a skin pathology, Rosacea (Egeberg et al., 2016), reinforces the idea that this compound is a promising candidate for PD pharmacological treatment.

The beneficial effect of Doxy in neurodegenerative disorders is not restricted to neuroprotection. Recently, our group has described that Doxy reduces AIMs manifestation in a LID rat model, where 6-OHDA lesioning of the medial forebrain bundle results in a severe and widespread loss of dopaminergic nerve endings within the entire striatum (Bortolanza et al., 2021). The anti-dyskinetic effect of Doxy was accompanied by the repression of striatal markers of neuroinflammation and oxidative stress, suggesting that this tetracycline operates *via* its anti-inflammatory activity (Bortolanza et al., 2021).

In this study, we attempted to further characterize Doxy anti-dyskinetic effects by using a mouse PD/LID model where the extent of striatal dopaminergic denervation is partial, i.e., either moderate or intermediate (Grealish et al., 2010; Dos-Santos-Pereira et al., 2016) and not widespread as it is in the PD rat model of extensive striatal denervation (Pavon et al., 2006; Dos-Santos-Pereira et al., 2016; Bortolanza et al., 2021). Our goals were more specifically to 1) determine whether Doxy reduces AIM manifestations in the groups of mice with moderate and intermediate dopaminergic lesions, 2) demonstrate that Doxy does not impair l-DOPA mediated locomotor improvement in lesioned mice, 3) exclude that neurorestorative effects of Doxy could contribute to its anti-dyskinetic effects, and 4) evaluate the impact Doxy on a battery of parameters associated with LID pathophysiology in the denervated striatum, including the transcriptional regulator Fos-B, the enzyme COX-2 and its active downstream product prostaglandin E2 (PGE_2_), as well as the pro-inflammatory cytokines TNF-α, IL-1β, and IL-6.

## 2 Material and methods

### 2.1 Drugs

6-Hydroxydopamine hydrochloride (6-OHDA; 2.5 mg ml^−1^ or 3.75 mg ml^−1^ diluted in saline 0.9% + 0.02% ascorbic acid), l-DOPA hydrochloride (25 mg.kg^−1^ i. p. + Benserazide 10 mg.kg^−1^ i. p. diluted in saline 0.9%), and doxycycline Hyclate (Doxy; 20 and 40 mg.kg^−1^ s. c. diluted in saline 0.9%) were obtained from Sigma-Aldrich, United States. The vehicle (Veh) control group received saline 0.9%.

### 2.2 Animals and 6-OHDA lesions

Eighty-five adult male C57⁄BL6 mice (FMRP-USP, Ribeirão Preto, Brazil; 20–25 g body weight) were housed under a 12-h light/dark cycle with free access to food and water. Male mice were preferentially used because the risk of developing PD is twice higher in men than in women (Cerri et al., 2019). All experiments were carried out under institutional approval of the Animal Care and Use Committee of the University of São Paulo (CEUA 026/2013), following the Brazilian Law nº 11.794/2008 and the Guide for the Care and Use of Laboratory Animals. All efforts were made to minimize animal suffering.

Mice were submitted to stereotaxic surgery for unilateral microinjection of 6-OHDA based on a protocol from a previous study (Dos-Santos-Pereira et al., 2016). The animals were anesthetized with 2,2,2- tribromoethanol (250 mg.kg^−1^ i. p.) and fixed into the stereotaxic apparatus for performing the surgery (David Kopf, model United States). 6-OHDA-HCl was diluted at 2.5 mg ml^−1^ or 3.75 mg ml^−1^ in 0.9% saline containing 0.02% ascorbic acid to produce animals with low to mild or intermediate LID manifestations, respectively. 6-OHDA was microinjected at 0.5 μL min^−1^ in a total volume of 2 μL per injection. Two sites of microinjection were used in the dorsolateral region of the striatum using the following stereotaxic coordinates (in mm from Bregma): anteroposterior (AP) = +0.5; lateral-lateral (LL) = +2.3 and dorsoventral (DV) = −3.9 (first injection) and −3.0 (second injection). After each microinjection, the cannula remained in place for five additional minutes to prevent the reflux of the injected solution. At the end of the surgical procedure, the animals were kept warm under a 60 W light bulb until complete recovery from anesthesia and then led to the vivarium, where they received amoxicillin (5 mg ml^−1^ orally) for 5 days.

### 2.3 l-DOPA treatment and experimental groups

#### 2.3.1 Inclusion criteria for LID assessment

**FIGURE 1:**
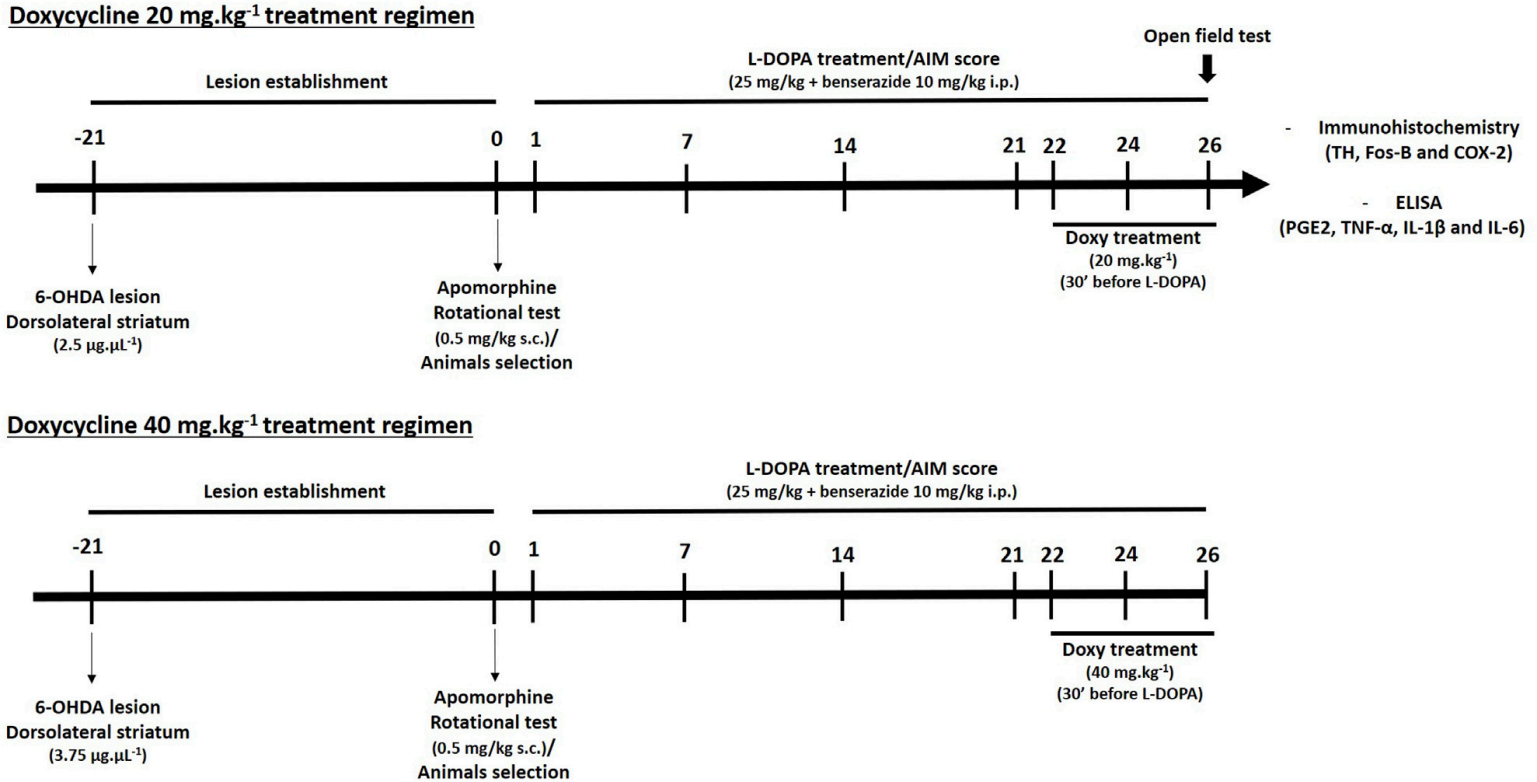
Schematic drawing of Doxy pharmacological treatments in hemiparkinsonian mice with established LID. Eighty-five mice were selected after 6-OHDA stereotaxic surgery (solutions of 2.5 or 3.75 µg µl^−1^ of 6-OHDA were used for Doxy 20 mg kg^−1^ or 40 mg kg^−1^ treatment groups, respectively) employing the apomorphine rotational test (0.5 mg.kg-1 s. c.). These mice were then treated chronically with l-DOPA for 21 days (25 mg kg^−1^ + benserazide 10 mg kg^−1^ i. p. once/day) and scored for AIMs. Once AIMs had been evaluated, hemiparkinsonian dyskinetic mice were divided into Veh- and Doxy (20 and 40 mg.kg^−1^ s. c.)-treated groups with equivalent AIMs scores. Then, a sub-chronic treatment with Doxy + L-DOPA or Veh + L-DOPA was administered for the next 5 days. During this period, mice were scored for AIMs on days 1, 3, and 5.

Twenty-one days after surgery for 6-OHDA lesioning, the animals were behaviorally assessed in the apomorphine rotational test (0.5 mg.kg^−1^ s. c.). We selected 6-OHDA lesioned animals that presented >3 contralateral rotations/min during the 45 min of the test (Iancu et al., 2005). This set of animals was chronically submitted to l-DOPA treatment, which consisted of one injection per day of l-DOPA (25 mg kg^−1^, i. p. + Benserazide 10 mg kg^−1^, i. p.) for 21 consecutive days. After the initial phase of chronic treatment with l-DOPA, mice with high levels of LID were selected [inclusion criteria: at least 40% of maximum AIM scores (Dos-Santos-Pereira et al., 2016)] and divided into the experimental groups described thereafter. Eleven animals that did not reach these criteria were excluded from the study. The timeline scheme for various treatments is presented in Figure 1.

#### 2.3.2 Experimental groups for LID assessment

**FIGURE 2:**
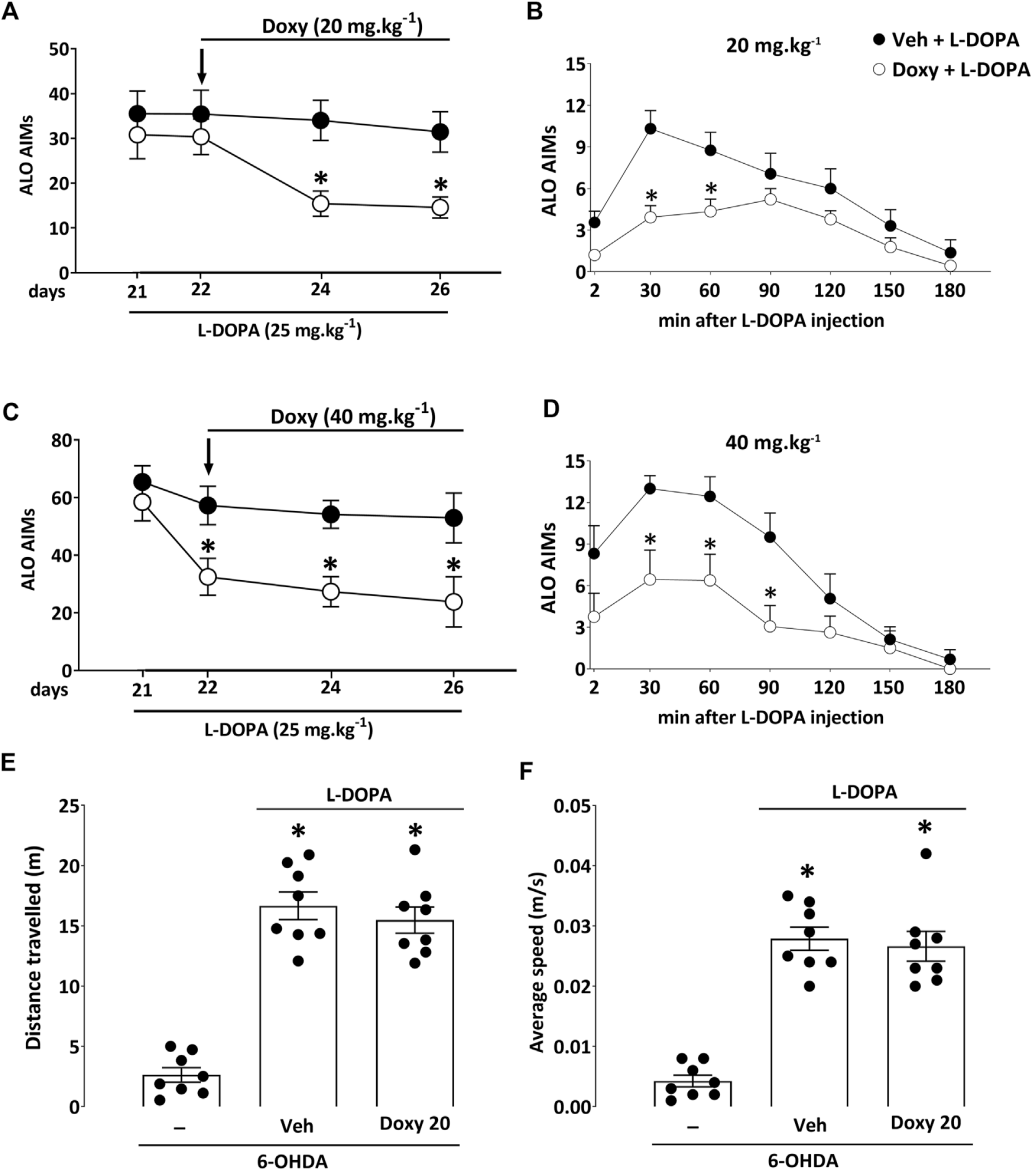
Impact of sub-chronic treatment with Doxy on established AIMs and locomotor activity in hemiparkinsonian mice with partial lesions. **(A)** AIMs observed in hemiparkinsonian mice with mild striatal lesions were significantly reduced at days 3 and 5 of sub-chronic treatment with Doxy (20 mg.kg^−1^ s. c.). **(B)** At day 5 of treatment with 20 mg kg^−1^, AIMs were significantly reduced 30 and 60 min after initiating l-DOPA treatment. **(C)** When given at 40 mg.kg^−1^ s. c. to hemiparkinsonian mice with intermediate striatal lesions, Doxy effectively reduced AIMs from day 1 of treatment until day 5. **(D)** At day 5 of treatment with 40 mg kg^−1^, AIMs were significantly reduced 30, 60, and 90 min after initiating l-DOPA treatment. **(E)** The total distance traveled and **(F)** the average speed of lesioned mice were increased by l-DOPA, and the co-administration of Doxy did not modify l-DOPA’s beneficial effects. a–d: *p < 0.05 vs. Veh (Data represent the mean ± SEM; r-MANOVA followed by Bonferroni post-hoc test, *n* = 8/group). e–f: *p < 0.05 vs 6-OHDA (Data represent the mean ± SEM; One way-ANOVA followed by Bonferroni post-hoc test, *n* = 8/group).

**FIGURE 3:**
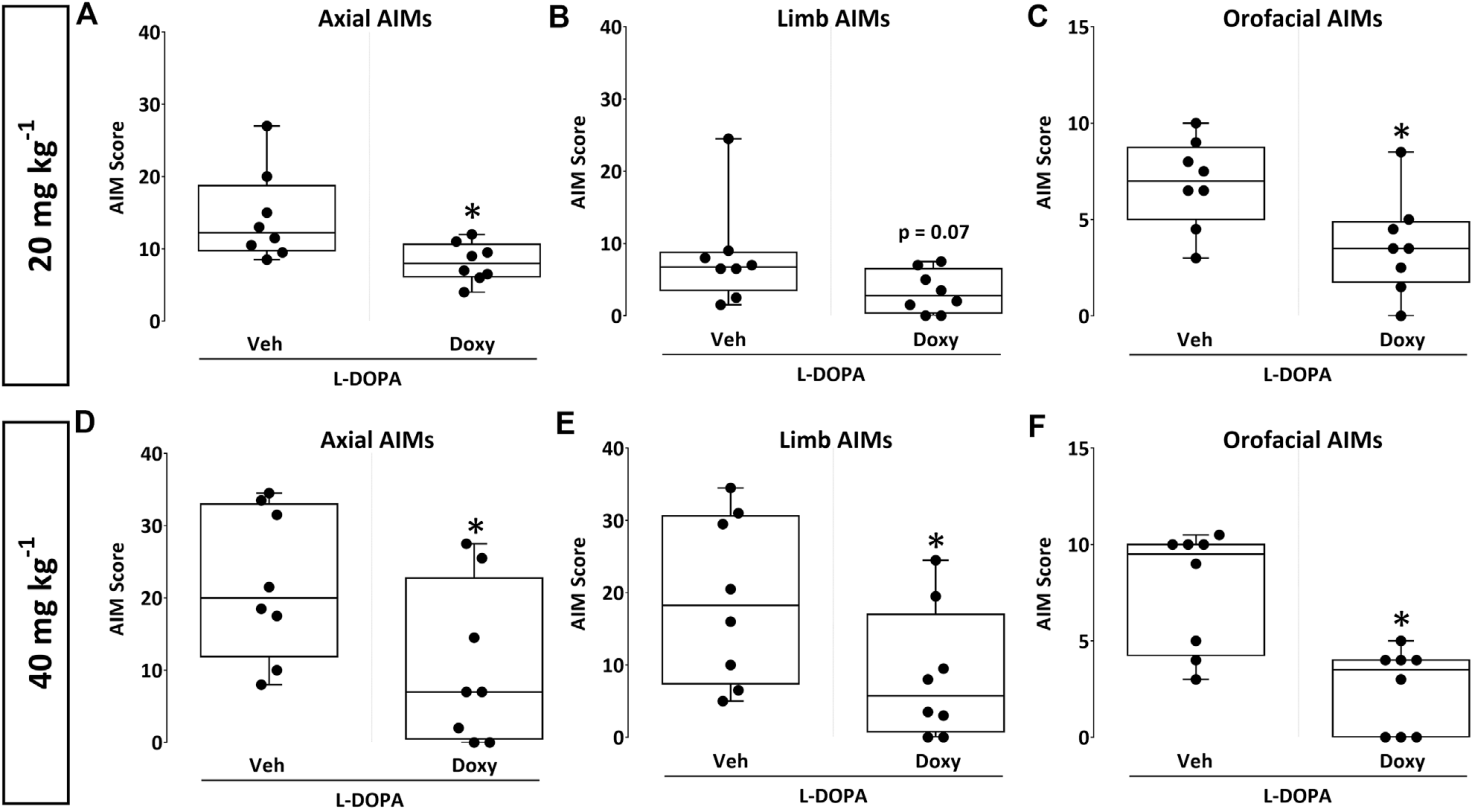
Box plot charts showing the impact of treatment with 20 and 40 mg kg^−1^ of Doxy on specific subtypes of AIMs. **(A–C)** Impact of Doxy 20 mg kg^−1^ on axial **(A)** limb **(B)** and orofacial **(C)** AIMs compared to the Veh-treated group in hemiparkinsonian mice with moderate striatal lesions. **(D–F)** Impact of Doxy 40 mg kg^−1^ on axial **(D)** limb **(E)** and orofacial **(F)** AIMs compared to the Veh-treated group in hemiparkinsonian mice with intermediate striatal lesions. Box plots represent the summary of the dataset for each parameter (minimum, lower quartile, median, upper quartile, and maximum). *p < 0.05 vs. Veh, Mann-Whitney test, *n* = 8/group. Note that the *p*-value in b is close to significance (*p* = 0.07).

In the first experiment, 16 dyskinetic mice with moderate dopaminergic lesions (∼50% nigrostriatal lesion) were sub-chronically exposed for 5 days to 20 mg kg^−1^ of Doxy s. c. or to vehicle (Veh) 30 min before l-DOPA treatment (*n* = 8/group; Figure 2A,B and 3A-C). In a second experiment, 16 dyskinetic mice with intermediate dopaminergic lesions (∼75% nigrostriatal lesion) were exposed for 5 days to 40 mg kg^−1^ of Doxy s. c. or to vehicle (Veh) before l-DOPA treatment (n = 8/group; Figure 2C,D and 3D-F). Each Veh group received saline 4 ml kg^-1^ s. c. LID was evaluated immediately after administration of l-DOPA on days 1, 3, and 5 of Doxy treatment (Figure 1).

#### 2.3.3 Experimental groups for locomotor activity evaluation with the open field test

An independent group of 24 parkinsonian mice treated with Doxy 20 mg kg^−1^ was evaluated for locomotor activity in the open field test to test whether Doxy possibly influenced the beneficial effects of l-DOPA in parkinsonian mice with reduced locomotor activity. We compared locomotor activities in groups of lesioned mice receiving DOXY/l-DOPA (*n* = 8), Veh/l-DOPA (*n* = 8), or 6-OHDA only (*n* = 8) (Figure 1).

### 2.4 Evaluation of l-DOPA-induced AIMs

l-DOPA-induced AIMs were evaluated as previously described (Dos-Santos-Pereira et al., 2016). Briefly, a trained observer aware of each group experimental treatment assessed each mouse for the presence of AIMs at 2, 30, 60, 90, 120, 150, and 180 min after administration of l-DOPA (or Veh). Axial, limb, and orolingual (ALO) AIMs subtypes were scored during 2-min periods for each analysis. The severity of AIMs three subtypes (axial, limb and orofacial) was rated ranging from 0 to 4 (where 0 = absent, 1 = occasional, 2 = frequent, 3 = continuous but interrupted by sensory distraction, and 4 = continuous, severe, and not suppressible). For axial and limb AIMs, amplitude scores (the maximum amplitude of the AIMs during the observation period) ranged from 0 to 4 (Bortolanza et al., 2016). During each monitored period, the sum of the severity x amplitude scores of axial, limb, and orolingual AIMs was determined as ‘ALO AIMs.’ Dyskinetic movements were expressed as a total score at each observation time point. Mean or median values represent the sum of all time points analyzed in the interval of 2–180 min for each experimental group, i.e., the global AIMs score (ALO AIMs). ALO AIMs scores were also plotted as a function of time post-l-DOPA administration (2, 30, 60, 90, 120, 150, and 180 min) to generate a dyskinesia time curve.

### 2.5 Open field test

Animals were submitted to the open field test 3 h after the last injection of l-DOPA. The open field consisted of a Plexiglas circular arena (40 cm diameter) with 40-cm-high walls. The animals were placed in the center of the arena, and the total distance traveled and immobility time were determined over 10 min using the ANYMAZE software (Stoelting Co., Ranelagh, Dublin, Ireland).

### 2.6 Immunohistochemistry

Striata of lesioned mice treated with 20 mg kg^−1^ of Doxy or vehicle were dissected, processed, and analyzed as previously described (Dos-Santos-Pereira et al., 2016). For immunodetection studies, we used tyrosine hydroxylase (TH, 1:2000; United States Biological, #T9237-13, lot L17071805), Fos-B/∆Fos-B (1:1000, Santa Cruz Biotechnology, #sc-48, lot J0814), and cyclooxygenase-2 (COX-2, 1:600; Cayman Chemical, #160106, lot 200,023–200024) antibodies. Quantitative analyses were then performed based on procedures previously described (Dos Santos Pereira et al., 2020).

### 2.7 Prostaglandin/cytokines levels quantification

The striata of 6-OHDA-lesioned animals receiving either Veh/Veh, Veh + l-DOPA, or 6-OHDA + Doxy 20 mg kg^−1^ + l-DOPA were analyzed. Brain tissue was recovered using an adult mouse brain slicer matrix (World Precision Instruments, Inc.; Sarasota, United States). Approximately 10 mg of the striatum dorsolateral area (AP: +0.62 to +0.5; ML: ±2.2 to ±2.4; DV: +3.0 to +3.5; relative to Bregma) were recovered for analysis. The tissue amount was equalized at a concentration of 50 mg ml^−1^ and homogenized in a lysis buffer containing 20 mM Tris-HCl pH 8.0, 137 mM NaCl, 10% glycerol together with protease (10% v/v; Sigma), and phosphatase (1 tablet/10 ml; Roche) inhibitors. After centrifugation at 4 °C (10,000 rpm for 10 min), the supernatant was recovered and immediately frozen at −80°C before further use. Samples were then diluted (1:10) to reach a final amount of 5 mg/well. We used sandwich ELISA kits to measure cytokines (TNF-α, IL-1β, and IL-6) and (PGE_2_) in the dorsolateral striatum of dyskinetic mice. Corresponding protocols have been previously described elsewhere (Dos Santos Pereira et al., 2020). Optical density measurements were performed using a SpectraMax M4 spectrophotometer (Molecular Devices, Sunnyvale, CA). Sample values were extrapolated from a four-parameter logistic curve fit (GraphPad Prism 8, GraphPad Software).

### 2.8 Statistical analysis

R-MANOVA, followed by Bonferroni’s post-hoc-test, was used to evaluate ALO AIMs using two independent variables (drug treatment and time). Data are expressed as mean ± SEM for these experiments. Mann-Whitney was performed to analyze AIMs by categories (axial, limb, and orofacial AIMs). Data are expressed as median ± interquartile range for these experiments, showing individual values for each animal. For ELISA quantification, data were evaluated by one-way ANOVA followed by Bonferroni’s post hoc test. For TH, Fos-B, and COX-2 immunohistochemistry, data were assessed by r-MANOVA followed by Bonferroni’s post-hoc test using treatment and lesion side as variables. Data are expressed as mean ± SEM. The minimum level of significance was set at *p* < 0.05. All statistical analyses were performed using GraphPad Prism 8.0. The values of ANOVA statistics are presented in Supplementary Table 1.

## 3 Results

### 3.1 Doxy reduces LID in hemiparkinsonian mice with partial dopaminergic lesions

Chronic treatment of 6-OHDA-lesioned mice with l-DOPA induced a transient increase in axial, limb, and orofacial (ALO) AIM scores over time in mice with mild and intermediate dyskinetic signs (Figures 2A,C). A sub-chronic treatment with Doxy (20 mg.kg^−1^ s. c.) in the dyskinetic mice with mild l-DOPA-induced AIMs (2.5 mg ml^−1^ 6-OHDA; ∼50% nigrostriatal lesion) significantly reduced already established AIMs. AIMs were not diminished immediately but after three consecutive days of Doxy treatment compared to the Veh group. The inhibitory effect persisted until at least day 5 of Doxy treatment, as indicated by Bonferroni’s post-hoc analysis (*p* < 0.05; Figure 2A). After 5 days of Doxy treatment, AIMs were reduced by > 50%. The time-course analysis of AIMs at day 5 of Doxy treatment over a period of 180 min revealed that Doxy leads to a substantial decrease of LID 30 and 60 min after l-DOPA treatment (*p* < 0.05; Figure 2B) but not at later stages of AIMs manifestations.

To demonstrate that dyskinetic mice with more robust AIMs still respond favorably to Doxy, we challenged the group of mice with intermediate striatal lesions (3.75 mg.ml^−1^ 6-OHDA; ∼75% nigrostriatal lesion) with a higher dose of the tetracycline, i.e., 40 mg kg^−1^. In this paradigm, AIMs were already reduced after Doxy’s first administration, and subsequent Doxy injections produced even slightly better inhibitory effects (*p* < 0.05 for all days of Doxy treatment vs Veh). At day 5 of Doxy treatment, the 40-mg.kg^−1^ dose of Doxy reduced AIMs by 65% compared to the Veh group (Figure 2C). The time-course analysis of AIMs at day 5 of Doxy treatment over a period of 180 min showed a substantial decrease of LID 30, 60, and 90 min after l-DOPA treatment (*p* < 0.05; Figure 2D).

Note that l-DOPA strongly increased locomotor activity (distance traveled; average speed) in parkinsonian mice with mild lesions compared with untreated ones (*p* < 0.05 vs 6-OHDA) and co-treatment with 20 mg kg^−1^ Doxy did not reduce the promotor effect of l-DOPA treatment on locomotion (*p* > 0.05 vs Veh) (Figures 2E,F).

### 3.2 Impact of Doxy on different subtypes of AIMs in hemiparkinsonian mice with partial dopaminergic lesions

We analyzed the effects of Doxy on individual scores for each AIM subtype at day 5 of Doxy treatment (Figure 3A-F). We observed that Doxy at 20 mg kg^−1^ reduced axial and orofacial (Figure 3A and 3C; *p* < 0.05) AIMs in mice with mild lesions, demonstrating the effectiveness of this compound on these two types of involuntary movements. The effects of 20 mg kg^−1^ Doxy on limb AIMs were just above statistical significance (*p* = 0.07) (Figure 3B). Dyskinetic animals with intermediate striatal lesions receiving Doxy at a dose of 40 mg kg^−1^ displayed a significant reduction of axial, limb, and orofacial AIMs scores (*p* < 0.05 vs Veh) (Figure 3D-F).

### 3.3 Anti-dyskinetic effects of doxy do not depend on potential neurorestorative effects in lesioned mice

**FIGURE 4:**
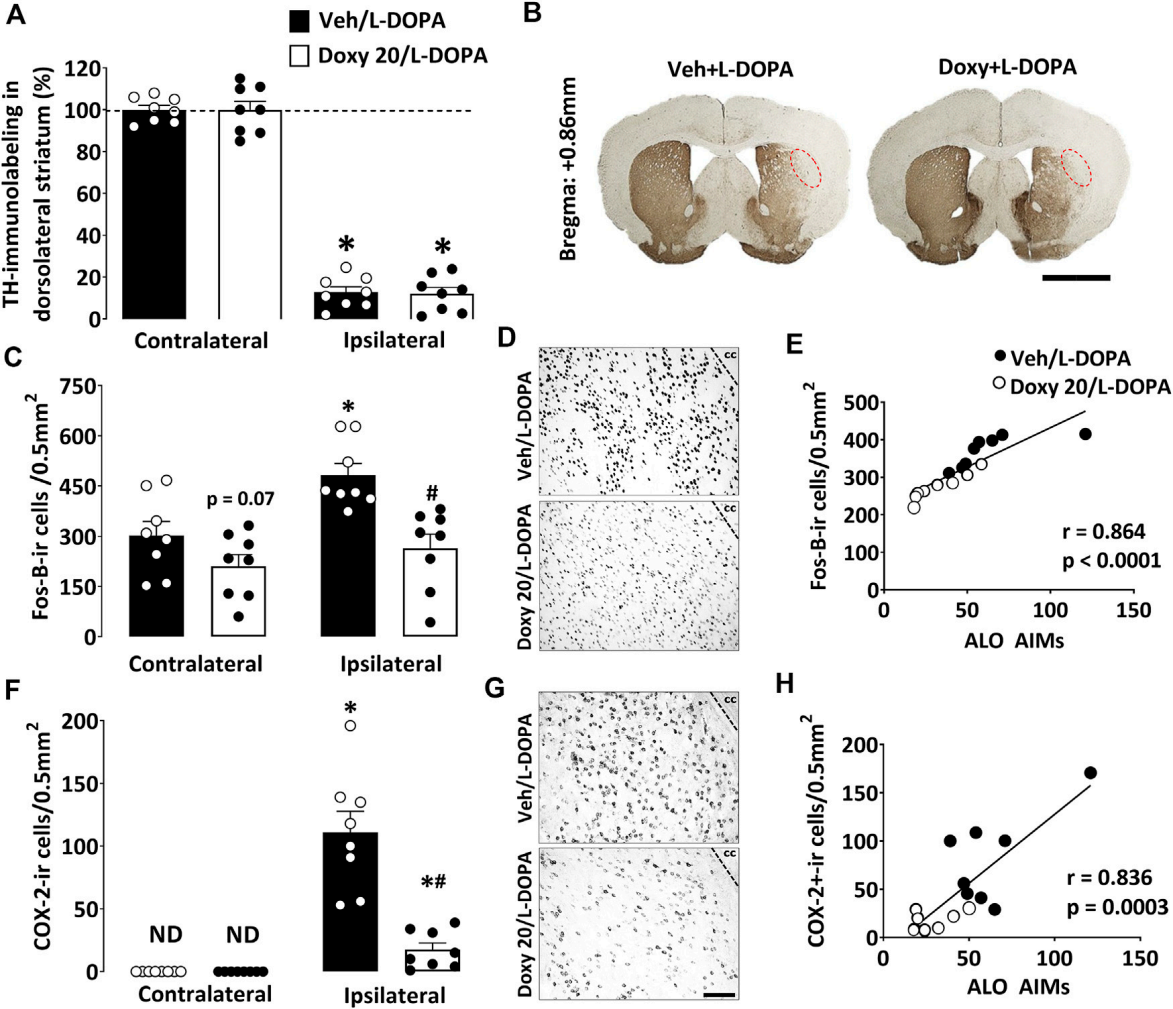
Analysis of the impact of Doxy treatment on TH, Fos-B, and COX-2 expression within the dorsolateral striatum of mice with partial lesions. **(A)** Quantitative optical density of striatal TH immunolabelling in coronal sections of the striatum of lesioned mice treated or not with 20 mg kg^−1^. **(B)** Photomicrographs of coronal sections of the rostral striatum illustrate that the loss of TH immunosignal is limited to the dorsolateral area of the striatum. **(C)** Chronic treatment with l-DOPA increased Fos-B expression in the lesioned mice’s dorsolateral area of ​​the striatum, and co-treatment with 20 m kg^−1^ Doxy significantly reduced the number of Fos-B^+^ cells. **(D)** Representative photomicrographs of Fos-B-ir cells in the dorsolateral striatum of Veh/l-DOPA and Doxy/l-DOPA-treated mice. **(E)** Positive correlation between the intensity of AIMs and Fos-B expression in lesioned mice treated with Veh or Doxy + l-DOPA. **(F)** The number of COX-2^+^ cells increased mainly in the dorsolateral striatum of lesioned mice after l-DOPA treatment and co-treatment with 20 m kg^−1^ Doxy significantly reduced the number of these cells. **(G)** Representative photomicrographs describing COX-2-ir cells in the lesioned striatum of Veh/l-DOPA and Doxy 20/l-DOPA-treated animals. **(H)** Positive correlation between the intensity of AIMs and the number of COX-2^+^ cells in mice treated with Veh or Doxy + l-DOPA. a, c and **(F)** *p < 0.05 vs unlesioned side; Two-way ANOVA followed by Bonferroni analysis, n = 8/group. e and **(H)** Spearman correlation. Values ​​are presented as mean ± SEM. cc: corpus callosum. ND: non-detectable. The red dashed line area corresponds to the striatum portion in which TH, Fos-B, and COX-2 were quantified. Scale Bar = 1 mm **(B)** and 50 µm **(D–G)**.

We also compared the extent of the dopaminergic lesion in the dorsolateral striatum of mice with moderate dyskinetic signs treated or not with Doxy (Figure 4). A substantial reduction of the TH immunoreactive (ir) signal was observed when comparing the dorsolateral striata ipsilateral and contralateral to the lesion side (*p* < 0.05). The treatment with 20 mg kg^−1^ of Doxy did not influence the lesion size of 6-OHDA lesioned animals (Figures 4A,B) (*p* > 0.05), indicating that anti-dyskinetic effects of Doxy do not rely on potential restorative effects of this compound.

### 3.4 Doxy anti-dyskinetic effects are correlated to a reduction of Fos-B^+^ and COX-2^+^ cells in the dorsal striatum of partially lesioned mice

In mice with moderate dopaminergic lesions, we analyzed the expression of the classical histological marker of LID manifestations, the transcription factor Fos-B. The number of Fos-B-immunolabeled cells increased specifically in the dorsolateral striatum in dyskinetic mice (*p* < 0.05 vs contralateral side). Treatment with Doxy 20 mg kg^−1^ significantly reduced the expression of Fos-B^+^ cells in comparison to the Veh group (*p* < 0.05; Figures 4C,D). A significant correlation was found between the number of Fos-B^+^ cells in the dorsal part of the lesioned striatum and the intensity of AIMs displayed by parkinsonian mice (r = 0.864, *p* < 0.0001; Figure 4E).

The number of cells immunopositive for the COX-2 enzyme also increased specifically in the dorsolateral part of the lesioned striatum in dyskinetic mice with a moderate striatal lesion (*p* < 0.05 vs contralateral side), while co-treatment with Doxy led to a reduction of these cells (*p* < 0.05; Figures 4F,G). There was also a significant correlation between the number of COX-2 immunoreactive cells in the dorsolateral striatum and AIMs levels (r = 0.836, *p* = 0.0003; Figure 4H).

### 3.5 The reduction of l-DOPA-induced AIMs by doxy is correlated to the decrease of specific inflammatory markers in the dorsolateral striatum of partially lesioned mice

**FIGURE 5:**
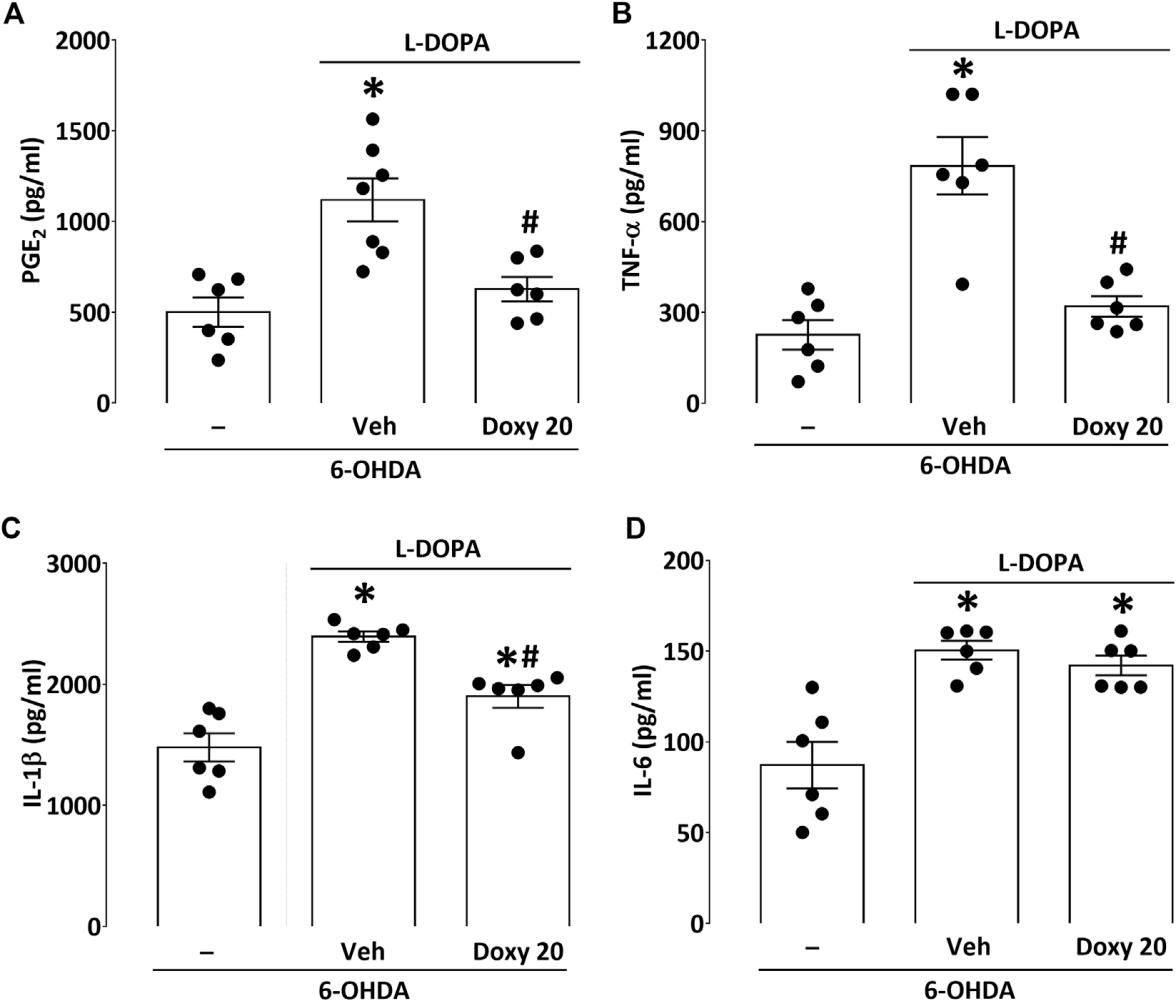
Effect of Doxy co-treatment on the production of PGE_2_ and pro-inflammatory cytokines in mice with partial lesions. **(A)** l-DOPA treatment increased PGE_2_ levels in the striatum of lesioned mice, and the co-treatment with Doxy 20 mg kg^−1^ suppressed this effect. **(B)** Doxy was also similarly effective in preventing the increase of TNF-α striatal levels induced by l-DOPA treatment. **(C)** Doxy only partially reduced the elevation of IL-1β levels observed after l-DOPA treatment. **(D)** The co-treatment with Doxy had no impact on the increase of IL-6 levels caused by l-DOPA treatment. *p < 0.05 vs 6-OHDA; #*p* < 0.05 vs Veh (One-way ANOVA followed by Bonferroni. *n* = 6/group). Values (pg/ml) are presented as mean ± SEM.

In this set of experiments, the effect of Doxy 20 mg kg^−1^ was reevaluated just before sacrifice to confirm efficacy of the treatment (Mann-Whitney U = 1.0, *p* = 0.0043; data not shown). In lesioned mice with mild dyskinetic signs, l-DOPA caused a significant elevation of the downstream product of COX-2, PGE_2_ (Figure 5A). Interestingly, PGE_2_ levels returned to control values in Doxy-treated mice (*p* < 0.05 vs Veh; Figure 5A). Similarly, Doxy treatment totally prevented the increase of TNF-α observed in the striatum of dyskinetic mice (*p* < 0.05 vs Veh; Figure 5B), while it partially reduced the elevation of IL-1β (*p* < 0.05 vs Veh; Figure 5C). At variance, Doxy was ineffective against the increase of IL-6 observed after l-DOPA treatment (*p* > 0.05 vs Veh; Figure 5D).

## 4 Discussion

This study shows that Doxy reduces dyskinesia when used at doses of 20 and 40 mg kg^−1^ in mice with moderate and intermediate striatal dopaminergic lesions, respectively. At 20 mg kg^−1^, Doxy affected axial and orofacial AIMs, whereas, at 40 mg kg^−1^, Doxy effectively diminished the three subtypes of AIMs (axial, limb, and orofacial). Using the dose of 20 mg kg^−1^, we showed that Doxy did not compromise l-DOPA-dependent improvement of locomotor activity observed in the lesioned mice. Besides, Doxy did not modify the intensity of the striatal tyrosine hydroxylase immunosignal, thus excluding a potential neurorestorative effect of the tetracycline on dopaminergic nerve terminals. Most interestingly, the treatment with 20 mg kg^−1^ of Doxy repressed a number of striatal markers of inflammation, comforting the view that the anti-dyskinetic effects of this tetracycline result from its immunosuppressive action on ongoing neuroinflammatory processes in the denervated striatum.

### 4.1 Doxy works as an anti-dyskinetic drug in mice with mild and intermediate dopaminergic lesions

Our present data further comfort the idea that Doxy acts as an effective anti-dyskinetic drug. Indeed, we demonstrate that a sub-chronic treatment with this tetracycline effectively decreases LID in parkinsonian mice with moderate or intermediate dopaminergic lesions. These data need to be put in perspective with a previous study showing that Doxy diminishes AIMs in a parkinsonian rat model with severe dopaminergic lesions that extend to the entire striatum (Bortolanza et al., 2021). Thus, it is clear that Doxy can produce anti-dyskinetic effects in rats and mice, suggesting that the effects of this compound are not species-specific. Besides, Doxy effects remain observable with striatal lesions ranging from partial and intermediate to severe. Doxy, at 20 mg kg^−1^, seems to be effective in diminishing LID in mice with moderate lesions, whereas a dose of 40 mg kg^−1^ appears to be required to inhibit LID manifestations in either mice or rats where dopaminergic neurodegeneration is more extensive (Bortolanza et al., 2021).

It should also be noted that the treatment with the dose of 20-mg.kg^−1^ of Doxy suppressed LID manifestation only after 3 days of treatment in mice with moderate dopaminergic lesions. Nevertheless, the anti-dyskinetic effect persisted thereafter if the treatment with Doxy was repeated. This indicates that the pathogenic mechanism associated with dyskinesia is repressed by the lowest dose of Doxy but obviously less rapidly than with the highest dose, which results in immediate anti-dyskinetic effects when administered just before l-DOPA. Future studies should tell us how quickly the anti-dyskinetic effects of Doxy wear off if the administration of l-DOPA is repeated while Doxy treatment discontinued.

### 4.2 Doxy does not impair locomotion improvement by l-DOPA in partially lesioned mice

Note that mice with mild lesions were hypokinetic, as attested by a reduction in the distance they traveled and the drop in their average speed. Most importantly, the improvement of the hypokinetic behavior of those mice by l-DOPA was not affected by the dose of Doxy of 20 mg kg^−1^, which is effective against dyskinesia, suggesting that Doxy has the pharmacologic profile of anti-dyskinetic medication for PD patients.

In keeping with that, a Brazilian proof-of-concept pilot study has proven that eight PD patients under l-DOPA treatment that received Doxy 200 mg. day^−1^ for 12 weeks had an attenuation on the Dyskinesia Rating Scale total score as well as a reduction on the on-time with troublesome LID without worsening parkinsonism (Santos-Lobato et al., 2022). This indicates that results obtained with Doxy in PD rodent models of LID are quite predictive of the effects of this compound in the human pathology.

### 4.3 Is doxy eliciting anti-dyskinetic effects at an antimicrobial dose?

Because a prolonged treatment with antibiotic doses of Doxy could result in a number of side effects, in particular by interfering with the host microbiome, we wished to determine whether the 20-mg.kg^−1^ dose of Doxy effective against LID also exerts antimicrobial effects. Even if we can only speculate on that matter, some arguments suggest that this dose yields plasma concentrations above minimal inhibitory concentrations estimated between 0.1–0.5 μg ml^−1^ for sensitive bacterial strains (Vargas-Estrada et al., 2008; Castro et al., 2009). Still consistent with this hypothesis, Lucchetti et al. (2019) showed that intraperitoneal injections of 10 mg kg^−1^ of Doxy produce peak concentrations of 2 μg ml^−1^ in the plasma of young adult male C57BL/6 mice. Similar plasma peak values of about 3 μg ml^−1^ were obtained in rats receiving 10 mg kg^−1^ of Doxy administered subcutaneously (Vargas-Estrada et al., 2008). These values are superimposable to data in humans, as individuals undergoing Doxy treatment with the usual antibiotic treatment regimen of 200 mg. day^−1^ *per os* have plasma concentrations ranging from 2.6 to 5.9 μg ml^−1^ (Saivin and Houin, 1988; Binh et al., 2009; Montagna et al., 2013). Overall, this means that the lowest dose of Doxy (i.e., 20-mg.kg^−1^) effective against LID probably has antimicrobial effects. Note that PD patients with LID responded positively to Doxy, at a dose of 200 mg. day^−1^, which has antibiotic action (Santos-Lobato et al., 2022).

Doxy prolonged treatment can be responsible for substantial side effects on microbiota, such as gastrointestinal discomfort, diarrhea, and dizziness (Walker et al., 2005; Gu et al., 2012; Bortolanza et al., 2018). Thus, doses that do not cause such side effects would be ideal for chronic therapy in PD patients with LID. Because PD is a disease that primarily affects elderly people, the use of Doxy in this category of the population also needs to be investigated appropriately. It is essential to consider that, due to different absorption, distribution and metabolism, aging patients can present a completely different response to chronic tetracycline treatment (Giarratano et al., 2018).

### 4.4 LID inhibition by doxy is not related to neuroprotection

The number of studies pointing to the neuroprotective potential of tetracyclines, including Doxy, has continued to expand in recent years (Bortolanza et al., 2018). Doxy crosses the blood-brain barrier (Andersson and Alestig, 1976), reaching brain areas where neurodegeneration is prominent. Neuroprotection by Doxy has been demonstrated in preclinical models of prion disease, Alzheimer’s disease, multiple sclerosis, and PD (Cho et al., 2009; Griffin et al., 2011; Lazzarini et al., 2013; Santa-Cecilia et al., 2019; Amaral et al., 2021; Dominguez-Meijide et al., 2021).

Yet, neuroprotective/neurorestorative effects cannot account for the efficacy of Doxy against LID in the present rodent PD model. Indeed, the TH immunosignal was similar in the striatum of lesioned mice treated or not treated with Doxy 20 mg kg^−1^, which indicates that the density of dopaminergic nerve endings remains unchanged after Doxy treatment. Besides, the fact that the 40 mg kg^−1^ dose of Doxy had an immediate inhibitory effect on AIMs also rules out a possible restorative effect on dopaminergic nerve terminals. Therefore, Doxy seems to operate here by preventing a mechanism that is downstream to dopaminergic neurodegeneration and rapidly inhibitable.

### 4.5 Doxy prevents the induction of Fos-B and the activation of neuroinflammatory processes in the denervated striatum

It is well established that LID pathophysiology results from elevated, inconsistent stimulation of striatal dopamine and glutamate receptors in the striatum that leads to sustained downstream modifications in the expression of several transcription factors or signaling molecules (Cenci et al., 1998; Konradi et al., 2004; Cenci and Lundblad, 2006; Huot et al., 2013). Among them, the transcription factor Fos-B may participate in the mechanisms of altered neuronal responses to dopamine that generate LID (Beck et al., 2019). Precisely, Fos-B was found to accumulate in subpopulations of striatal medium spiny neurons positive for calbindin and nitric oxide synthase in rodents and non-human primates with LID (Pavon et al., 2006; Feyder et al., 2011; Padovan-Neto et al., 2015; Beck et al., 2019). In line with these observations, we found that repeated administration of l-DOPA resulted in a substantial increase in the number of Fos-B-ir cells in the denervated striatum of lesioned mice. At the dose of 20 mg kg^−1^, Doxy prevented l-DOPA-mediated Fos-B induction indicating that the reduction of AIMs by Doxy was somehow related to its repressive action on this immediate-early gene.

Similarly, this dose of Doxy prevented the elevation of the pro-inflammatory enzyme COX-2 and its downstream product PGE_2_, which is a potent inflammatory mediator (Johansson et al., 2015). This suggests that Doxy might repress a mechanism by which Fos-B controls COX-2-mediated PGE_2_ production in the denervated striatum of parkinsonian mice. However, Cervantes-Madrid and others (2017) suggested that Fos-B plays a role in regulating COX-2 expression in colorectal cancer cells, but they failed to show that Fos-B knockout affects PGE_2_ levels in these cells.

The onset and progression of LID are also thought to depend on other pro-inflammatory factors, such as cytokines released by activated glial cells. This is the case in both rodents (Del-Bel et al., 2016; Carta et al., 2017; Pisanu et al., 2018; Junior et al., 2020) and non-human primates (Morissette et al., 2022). In the present study, we show that the 20-mg.kg^−1^ dose of Doxy totally prevented the rise in striatal TNF-α observed in dyskinetic mice and only partially that of IL-1β. Doxy had, however, no impact on the rise of IL-6 levels. These results indicate that the anti-dyskinetic effects of Doxy may result from its immunosuppressive effects on TNF-α and IL-1β. The inhibitory effects on TNF-α may be particularly important as this cytokine has the potential to stimulate the release of glutamate, a neurotransmitter crucially involved in dyskinesia (Dos Santos Pereira et al., 2020). It is also interesting to note that both TNF-α and IL-1β can induce COX-2 (Nakao et al., 2002; Neeb et al., 2011), which is elevated in the denervated striatum of dyskinetic mice. Finally, current observations also align with previous reports showing that Doxy exerts anti-inflammatory effects on brain glial cells (Ferreira Junior et al., 2021; Lazzarini et al., 2013; Santa-Cecilia et al., 2016; Pereira et al., 2021).

## 5 Conclusion

The present work provides further information on how Doxy may reduce LID in the context of PD neurodegeneration. Specifically, we show that Doxy may operate by inhibiting the activation of several neuroinflammatory processes that may mutually influence each other to create a dyskinetic state upon l-DOPA treatment. Because Doxy is a well-known and widely used tetracycline with a safe toxicological profile, we further suggest that it may be used as an adjunct treatment to l-DOPA in PD patients that start to develop LID.

Role of the funding source: This project was carried out in the framework of the CAPES-COFECUB program between the French and Brazilian research institutions (project 88,887.192409/2018–00 - Me928/19). The following Brazilian agencies provided financial support - Coordenação de Aperfeiçoamento de Pessoal de Nível Superior (CAPES; PROEX0051047 - USP/RP), Fundação de Amparo à Pesquisa do Estado de São Paulo (FAPESP 2017/24,304–0; 2017/14,207–7) and Conselho Nacional de Desenvolvimento Cientifico e Tecnológico (CNPq; 201,187/2016–7). The French Agency France Parkinson (DOXYPARK) provided financial support for the French team.

## Data Availability

The raw data supporting the conclusions of this article will be made available by the authors, without undue reservation.
