# Supplementary material for: Doxycycline attenuates l-DOPA-induced dyskinesia through an anti-inflammatory effect in a hemiparkinsonian mouse model
Source: Front Pharmacol. 2022 Nov 23;13:1045465. doi: 10.3389/fphar.2022.1045465 (PMC9728610; doi:10.3389/fphar.2022.1045465)
Supplement: Supplementary file 1 [file Table1.doc]

**Supplementary Table –** Statistical analysis performed in this study.

- Group characteristics are properly identified throughout the text or in figure 1
- The number of individual assets used in each group is described in figure legends

| **Figure** | **Parameter** | **Test** | **Value** |
| --- | --- | --- | --- |
| **Figure 2A** | Global Axial, Limb and Orofacial abnormal involuntary movements score  *Groups: Veh + L-DOPA vs. Doxy 20 + L-DOPA* | Two-way ANOVA for repeated measures (time) followed by Bonferroni’s multiple comparison’s test | Time: **F_3,39_ = 11.44**  **p < 0.0001**  Treatment: **F_1,13_ = 3.95**  **p = 0.06**  Interaction: **F_3,39_ = 5.55**  **p = 0.002** |
| **Figure 2B** | Time-course of Axial, Limb and Orofacial abnormal involuntary movements score  *Groups: Veh + L-DOPA vs. Doxy 20 + L-DOPA* | Two-way ANOVA for repeated measures (time) followed by Bonferroni’s multiple comparison’s test | Time: **F_6,68_ = 52.13**  **p < 0.0001**  Treatment: **F_1,13_ = 4.59**  **p = 0.05**  Interaction: **F_6,68_ = 8.15**  **p < 0.0001** |
| **Figure 2C** | Global Axial, Limb and Orofacial abnormal involuntary movements score  *Groups: Veh + L-DOPA vs. Doxy 40 + L-DOPA* | Two-way ANOVA for repeated measures (time) followed by Bonferroni’s multiple comparison’s test | Time: **F_3,39_ = 9.03**  **p < 0.0001**  Treatment: **F_1,13_ = 8.82**  **p = 0.01**  Interaction: **F_3,39_ = 2.03**  **p = 0.12** |
| **Figure 2D** | Time-course of Axial, Limb and Orofacial abnormal involuntary movements score  *Groups: Veh + L-DOPA vs. Doxy 40 + L-DOPA* | Two-way ANOVA for repeated measures (time) followed by Bonferroni’s multiple comparison’s test | Time: **F_6,68_ = 27.8**  **p < 0.0001**  Treatment: **F_1,13_ = 5.49**  **p = 0.03**  Interaction: **F_6,68_ = 3.78**  **p = 0.002** |
| **Figure 2E** | Distance travelled (m)  *Groups: 6-OHDA vs. Veh + L-DOPA vs. Doxy 40 + L-DOPA* | Ordinary One-way ANOVA followed by Bonferroni’s test | **F_2,21_ = 63.76**  **p < 0.0001** |
| **Figure 2F** | Average speed (m/s)  *Groups: 6-OHDA vs. Veh + L-DOPA vs. Doxy 40 + L-DOPA* | Ordinary One-way ANOVA followed by Bonferroni’s test | **F_2,21_ = 48.97**  **p < 0.0001** |
| **Figure 3A** | Axial abnormal involuntary movements score  *Groups: Veh + L-DOPA vs. Doxy 20 + L-DOPA* | Mann Whitney test | **U: 9.5**  **p = 0.01** |
| **Figure 3B** | Limb abnormal involuntary movements score  *Groups: Veh + L-DOPA vs. Doxy 20 + L-DOPA* | Mann Whitney test | **U: 15**  **p = 0.07** |
| **Figure 3C** | Orofacial abnormal involuntary movements score  *Groups: Veh + L-DOPA vs. Doxy 20 + L-DOPA* | Mann Whitney test | **U: 11.5**  **p = 0.03** |
| **Figure 3D** | Axial abnormal involuntary movements score  *Groups: Veh + L-DOPA vs. Doxy 40 + L-DOPA* | Mann Whitney test | **U: 12**  **p = 0.03** |
| **Figure 3E** | Limb abnormal involuntary movements score  *Groups: Veh + L-DOPA vs. Doxy 40 + L-DOPA* | Mann Whitney test | **U: 13**  **p = 0.04** |
| **Figure 3F** | Orofacial abnormal involuntary movements score  *Groups: Veh + L-DOPA vs. Doxy 40 + L-DOPA* | Mann Whitney test | **U: 7.5**  **p = 0.007** |
| **Figure 4A** | Tyrosine Hydroxylase immunoreactivity (Optical density)  *Groups: Veh + L-DOPA vs. Doxy 20 + L-DOPA (contralateral vs. ipsilateral)* | Two-way ANOVA for repeated measures (side) followed by Bonferroni’s multiple comparison’s test | Side: **F_1,28_ = 838.3**  **p < 0.0001**  Treatment: **F_1,28_ = 0.011**  **p = 0.91**  Interaction: **F_1,28_ = 0.011**  **p = 0.91** |
| **Figure 4C** | Number of Fos-B+ cells in dorsal striatum/0.5 mm²  *Groups: Veh + L-DOPA vs. Doxy 20 + L-DOPA (contralateral vs. ipsilateral)* | Two-way ANOVA for repeated measures (side) followed by Bonferroni’s multiple comparison’s test | Side: **F_1,28_ = 9.11**  **p = 0.005**  Treatment: **F_1,28_ = 16.2**  **p = 0.0004**  Interaction: **F_1,28_ = 2.70**  **p = 0.11** |
| **Figure 4E** | Correlation of striatal Fos-B+ cells and abnormal involuntary movements score | Spearman correlation | **r = 0.95**  **p < 0.0001** |
| **Figure 4F** | Number of Fos-B+ cells in dorsal striatum/0.5 mm²  *Groups: Veh + L-DOPA vs. Doxy 20 + L-DOPA (contralateral vs. ipsilateral)* | Two-way ANOVA for repeated measures (side) followed by Bonferroni’s multiple comparison’s test | Side: **F_1,28_ = 54.04**  **p < 0.0001**  Treatment: **F_1,28_ = 28.61**  **p < 0.0001**  Interaction: **F_1,28_ = 28.61**  **p < 0.0001** |
| **Figure 4H** | Correlation of striatal COX-2+ cells and abnormal involuntary movements score | Spearman correlation | **r = 0.71**  **p = 0.004** |
| **Figure 5A** | PGE_2_ concentration in dorsal striatum (mg/ml)  *Groups: 6-OHDA only vs. Veh + L-DOPA vs. Doxy 20 + L-DOPA* | Ordinary One-way ANOVA followed by Bonferroni’s test | **F_2,15_ = 12.31**  **p = 0.0006** |
| **Figure 5B** | TNF-α concentration in dorsal striatum (mg/ml)  *Groups: 6-OHDA only vs. Veh + L-DOPA vs. Doxy 20 + L-DOPA* | Ordinary One-way ANOVA followed by Bonferroni’s test | **F_2,15_ =21.36**  **p < 0.0001** |
| **Figure 5C** | IL-1β concentration in dorsal striatum (mg/ml)  *Groups: 6-OHDA only vs. Veh + L-DOPA vs. Doxy 20 + L-DOPA* | Ordinary One-way ANOVA followed by Bonferroni’s test | **F_2,15_ =26.13**  **p < 0.0001** |
| **Figure 5D** | IL-6 concentration in dorsal striatum (mg/ml)  *Groups: 6-OHDA only vs. Veh + L-DOPA vs. Doxy 20 + L-DOPA* | Ordinary One-way ANOVA followed by Bonferroni’s test | **F_2,15_ = 16.08**  **p = 0.0002** |
